# How to establish and use local diagnostic reference levels: an ESR EuroSafe Imaging expert statement

**DOI:** 10.1186/s13244-023-01369-x

**Published:** 2023-02-06

**Authors:** John Damilakis, Guy Frija, Boris Brkljacic, Eliseo Vano, Reinhard Loose, Graciano Paulo, Hugues Brat, Virginia Tsapaki, the European Society of Radiology

**Affiliations:** 1 School of Medicine, University of Crete, Iraklion, Crete, Greece; 2 Université de Paris, 12 Rue de L’École de Médecine, 75006 Paris, France; 3 School of Medicine, University of Zagreb, Šalata 3, 10000 Zagreb, Croatia; 4 Complutense University, Madrid, Spain; 5 Institute of Medical Physics, Hospital Nuremberg, Prof.-Ernst-Nathan-Str. 1, 90419 Nuremberg, Germany; 6 Medical Imaging and Radiotherapy Department, ESTESC-Coimbra Health School, Instituto Politécnico de Coimbra, Rua 5 de Outubro, S. Martinho Do Bispo, 3046-854 Coimbra, Portugal; 7 Institut de Radiologie de Sion, Rue du Scex 2, 1950 Sion, Switzerland; 8 Medical Physics, Konstantopoulio General Hospital, Nea Ionia, Greece; 9 European Society of Radiology (ESR), Am Gestade 1, 1010 Vienna, Austria

**Keywords:** Diagnostic reference levels, Optimisation, Radiation dosage

## Abstract

**Abstract:**

Although the Diagnostic Reference Levels (DRLs) have been shown to be an important tool for optimising patient radiation protection, there are still difficulties related to the methodology that should be used to establish and use local DRL values. This statement represents the current view of the EuroSafe Imaging ‘Clinical DRLs’ working group formed with the purpose to produce scientific and educational material on DRLs and promote the concept of local DRLs. Guidelines on how to establish and how to use local DRLs presented herein can be implemented using a multidisciplinary team approach. Local DRLs are easy to determine and implement and they reflect local equipment performance and local clinical needs. They can be updated more frequently than the national DRLs, especially if a dose management system is available. To establish local DRLs, a practical approach could be to collect a reasonable set, i.e., at least 20–30 procedures, of data for well-defined clinical indications and calculate the 3rd quartile values. The median values of the distribution can be set to define the ‘typical values’. The International Commission of Radiological Protection (ICRP) suggests setting ‘typical values’ for newer technologies that enable decreased amounts of radiation exposure for a similar level of image quality. Local DRLs should be similar or lower to the national DRLs. They could be higher only if the clinical benefits for some medical indications are fully explained and reported. Local DRLs may be used as a quality benchmark to track outliers and can be also used as alert values.

**Key points:**

- Guidelines on how to establish and use local DRLs are presented.
- Local DRLs are easy to determine and implement and can be updated frequently
- Additionally, local DRLs can be used to track outliers.

## Patient summary

The principle of optimisation is applied to protect patients from unnecessary levels of radiation exposure. Diagnostic Reference Levels (DRLs) have been recommended as an optimisation tool by the International Commission of Radiological Protection. DRLs indicate whether the dose to patients from an X-ray procedure or the amount of radiopharmaceuticals administered for imaging is unusually high or unusually low. They are implemented for modalities such as Computed Tomography and X-ray mammography and do not apply to individual patients.

## Introduction

Diagnostic Reference Levels (DRLs) were recommended as an optimisation tool by the International Commission of Radiological Protection (ICRP) in the early 1990s and introduced in the European legislation in 1997. During the first years, DRLs were established by a limited number of countries only for a standard-size adult patient and a few anatomical radiography and CT protocols. Since then, medical imaging has progressed at a fast pace. A vast spectrum of new medical imaging systems and techniques have been developed. The awareness level concerning patient radiation safety has increased considerably. While most examinations are performed safely, there are situations where optimisation is lacking. DRLs have been shown to be a valuable tool for optimising patient radiation protection during diagnostic procedures. Dose management systems (DMS) have also been launched to aid in the automatic collection and processing of various technical parameters including dose indicators (metrics). Consequently, the number of newly published or updated DRLs is growing fast, allowing the DRL concept to be further implemented not only in diagnostic imaging but also in fluoroscopically-guided interventional procedures (FGIP).

Nevertheless, there are still issues and difficulties related to the methodology that should be used to establish and use DRL values. Analysis of the EUCLID (European Study on Clinical Diagnostic Reference Levels for X-ray Medical Imaging) European Commission (EC) project data revealed that CT DRLs vary between centres or countries mainly due to different number of phases or different scanning lengths [1]. Some studies include all phases in the DRLs determination, while others consider only a single phase. Critical information, for example, about the examination protocol or the size of the CT dose index (CTDI) phantom, that is used to estimate volumetric CTDI, is not always provided. Image quality and diagnostic information is not always taken into account in DRL determination. Inconsistent terminology used in different studies can lead to misinterpretation of results. CT DRLs have been established in many countries for anatomical locations but rarely for clinical indications, which reflect much better the image quality requirements. For example, non-contrast CT for ureteral stone detection can be performed by using lower doses than those used in other abdominal procedures such as CT for diagnosis of appendicitis because detection of stones is affected less by noise than low-contrast tissues. DRLs in Nuclear Medicine have not been determined in a standardised way [2]. There is a lack of national and regional DRLs for FGIP and paediatric imaging examinations. Moreover, local DRLs have been established only in a few healthcare facilities and are not easily accessible for benchmarking. This can be partly attributed to the fact that information about the establishment and use of local DRLs is limited.

## Regulatory requirements and recommendations

The European Union Directive 2013/59/Euratom [3] defines DRLs as ”dose levels in medical radiodiagnostic or interventional radiology practices or, in the case of radio-pharmaceuticals, levels of activity, for typical examinations for groups of standard-sized patients or standard phantoms for broadly defined types of equipment”. Article 56 of the 2013 Directive obliges member states to establish, review and use DRLs to optimise radiation protection.

The establishment and use of DRLs is also a requirement of the international Basic Safety Standards published by the International Atomic Energy Agency [4]. Requirement 34 on the responsibilities of the government specific to medical exposure states that a set of DRLs should be established for medical exposures in medical imaging, including FGIP considering the need for adequate image quality. It is also mentioned that registrants and licensees shall ensure a review is conducted to determine whether corrective action is needed if typical doses exceed DRLs.

In the context of Council Directive 2013/59/Euratom, the EUCLID project investigated the feasibility of establishing DRLs based on clinical indication [1]. A EUCLID survey found that the majority of EU countries have DRLs. However, the quantity and utilisation of these DRLs vary considerably between countries. EUCLID showed that establishing clinical indication based DRLs is a task that can be accomplished. Recommendations are provided to address issues related to the lack of DRLs or the lack of proper use of DRLs.

In a recent publication, the ICRP clarifies terminology related to DRLs and provides information on the use of DRLs for FGIP and medical imaging examinations performed on paediatric patients [5]. Moreover, it proposes changes in the conduct of relevant surveys and stresses the importance of including information on the role of DRLs in education and training of healthcare personnel.

The European Guidelines on Diagnostic Reference Levels for Paediatric Imaging (PiDRL) project provided recommendations on how to establish and use DRLs for paediatric X-ray examinations [6]. This guidance suggests physical quantities that are considered feasible as DRL quantities for radiography, fluoroscopy, and CT, and provides lists of examinations for which paediatric DRLs should be determined. According to the PiDRL document, the primary focus should be on establishment of local DRLs. PiDRL guidelines have been endorsed by the leading European professional and scientific societies in the area of medical X-ray imaging, i.e., the European Society of Radiology (ESR), the European Society of Paediatric Radiology (ESPR), the European Federation of Radiographer Societies (EFRS) and the European Federation of Organizations for Medical Physics (EFOMP).

## How to establish local DRLs

Local DRLs have been defined by the ICRP “for a defined clinical imaging task, based on the 75th percentile value of the distribution of the appropriate DRL quantity in a reasonable number (e.g., 10–20) of X-ray rooms”. The suggested application is for “local use to identify X-ray units requiring further optimisation” [5].

It is expected to use the local DRLs when local equipment or techniques have enabled a greater degree of optimisation. Table 1 summarises the different types of DRLs, methods of derivation, and areas of application recommended by ICRP.

**Table 1:** Types of diagnostic reference levels (DRLs), methods of derivation, and areas of application (from ICRP-135 [1])

Term	Area and facilities surveyed	Value in distribution used to set DRL	Application
Typical values	Healthcare facility consisting of several X-ray rooms or a small number of facilities or single facilities linked to a new technique	Median value of the distribution, as there are insufficient data to use the third quartile	Local use to identify X-ray units requiring further optimisation
Local DRL	X-ray rooms within a few healthcare facilities (e.g., with at least 10–20 X-ray rooms) in a local area	Third quartile of median values for individual X-ray rooms	Local use to identify X-ray units requiring further optimisation
National DRL	Representative selection of facilities covering an entire country	Third quartile of median values for individual X-ray rooms or of national values	Nationwide to identify X-ray facilities where optimisation is Needed
Regional DRL	Several countries within one continent	Median values of distributions of national values or 75th percentile of distribution for representative selection of healthcare facilities throughout the region	Countries within region without a relevant DRL or for which national DRL is higher than regional value

However, in clinical practice, the new X-ray equipment or post-processing techniques usually occur (at least initially) in only one X-ray room and in addition to the use of “typical values” for patient dose indicators, it may be useful to calculate the third quartile to be considered as a “local DRL” for the clinical procedures performed with the new equipment. “Typical value” is also used by the ICRP and is defined as “the median value of the distribution of the dosimetric quantity for a clinical imaging procedure”. ICRP suggests setting “typical values” for newer technologies that enable decreased amounts of radiation to be used in achieving a similar level of image quality. Where no national DRL values exist, “local DRLs or typical values” might be introduced to assist the optimisation process further. It should be emphasised that DRLs should not be interpreted as normal dose values since an acceptable image quality can be achieved at levels much lower than DRLs. The "typical value" can be used as a guide to encourage further optimisation in a facility.

The local DRLs may be very useful for other hospitals installing the same or similar imaging technology (e.g., spectral CT systems or low dose interventional systems) to be used for the same clinical indications or clinical tasks. Once the new technology is installed in several hospitals, a national (or regional) DRL could be proposed as the third quartile of the median values of different hospitals (Table 1).

It should be noted that ICRP also states that “flexibility is necessary for procedures where few data are available (e.g., interventional procedures in paediatric patients), or where data are available from only one or a few centres” [5].

Local DRLs have been used in the last years to report patient dose indicators with small samples (e.g., in paediatrics) [7], according to body metrics (7), after installing X-rays systems with new imaging technology, or when national DRLs are still not available for some clinical indications [9–12].

The PiDRL project [6] recommends establishing local DRLs for emerging or increasing new practices, such as hybrid imaging when the CT is used for diagnostic purposes and paediatric cone-beam CT (CBCT) examinations. It is also suggested to use local DRLs to follow patient dose levels and to find out if there are any unexpected changes due to equipment malfunction, unauthorised change of the imaging practice or lack of sufficient training of new users.

Thus, the practical approach could be to collect a reasonable set (e.g., minimum of 20–30 procedures) of dosimetric data for well-defined clinical indications and to calculate median and 3rd quartile values. The EUCLID project recommends establishment of CT DRLs taking into account all phases, since they include information of the entire CT examination [1]. The updation of local DRLs is not an easy process and takes time. However, DMS provide the opportunity of a dynamic approach allowing a frequent updation of local DRLs. Median values would be used as “typical values”, and third quartile values as initial “local LDRs” for that imaging clinical indication.

## Benefits and advantages to use local DRLs

- Easy to calculate and implement locally at the hospitals.
- Not necessary to wait for updates of national DRLs (if they exist).
- Possibility to set different values for different technologies (including different post-processing).
- They reflect local equipment performances.
- They reflect local clinical needs.
- Possibility to set values for more clinical indications than for national DRLs.
- Local DRLs may be “dynamic” and updated more frequently than the national DRLs.
- After optimisation programmes in the hospital (e.g., focussed training for some imaging modalities), local DRLs can be easily updated.
- Comparison of patient dose indicators with local DRLs may be easier using DMS.

In most cases, local DRLs should be similar (or lower) to the national or regional (e.g., European) DRLs, if they exist, for the investigated clinical indications. In exceptional cases (with the appropriate justification), local DRLs could be higher than national DRLs if the clinical benefits for some medical indications are reported.

The term DRL is generally associated with national DRLs, which are an obligation in many countries due to legal regulations in radiation protection. A strength of local DRLs is their applicability to local scenarios, e.g. different technologies, different patient characteristics and diseases, or procedures for which no national DRLs exist. Due to differences in methods of data collection and different procedure names, DRLs can differ from each other at all levels. The term local DRLs can refer to a facility or a group of facilities in the same geographical area. The strength of local DRLs has several reasons. In most countries national DRLs are established for a limited number of procedures for all modalities, [13], which, however, covers the largest proportion of radiological procedures. As it is desirable to have DRLs for as many procedures as possible, local DRLs can be set up for all procedures, including those without national DRLs. Local equipment or techniques have enabled a greater degree of optimisation, so that a value below the corresponding national DRL can be implemented [5, 7].

Examples are CT scanners with or without iterative reconstruction or deep learning reconstructions. Here the DRLs of the different modality classes can significantly differ for the same procedure. Local DRLs should generally be lower than national DRLs but can in rare cases be higher for procedures with high complexity levels or specific patient groups. Establishing and using local DRLs for optimisation becomes much easier with DMS [14]. DMS automatically provide dosimetric data for each modality and type of procedure with 25%, 50%, 75% quartiles and mean and median values. Deviations from local DRLs due to technical or human errors can be quickly identified. A problem with using local DRLs is, often, the different use of procedure names and, therefore, limited regional or local comparability.

## How to use local DRLs

The European Basic Safety Standards Directive expresses the need of protecting the general population from avoidable radiation exposure and urges to improve radiation protection in Europe (3). Optimisation follows the ALARA (as low as reasonably achievable) principle and requires the adequate image quality needed for the medical imaging procedure. Once the local DRLs have been established, their practical use should be included in a sustainable and continuous quality assurance program, performed by a local optimisation team, including at least a radiologist, a medical physicist, and a radiographer (2,5).

Three levels of use could be considered:

- For each clinical indication, local DRLs may be used as a quality benchmark to track outliers. Although DRLs do not represent dose limits, they could be used as alert values based on clinical indication and according to patient habitus (BMI) (8) as well as reconstruction technology used. An arbitrary alert level, such as twice the median of the local DRL distribution, can be used to impose a qualitative high dose examination justification (5).

A standardised list of justifications may be used to enable retrospective data analysis (Table 2). This dose alert justification process is also helpful for training new team members to the local standards.

**Table 2:** List of dose alert justifications

**Dose alert justification**	
Acquisition length	A
Additional acquisition	B
Patient Body Mass Index	C
Patient Movement	D
Contrast media injection issue	E
Non-standardised protocol	F
Other	G

- Monthly, a simplified report with three performance indicators may represent a continuous educational tool for local teams [14], including:
  - tracking of unjustified high doses with a retrospective requirement of justification, enabling a progressive change management and a prospective process improvement,
  - analysing the percentage of non-standardised protocols, representative of practice uniformity in terms of number of series for the same clinical indication or even protocol type. High variability represents an opportunity to improve practice uniformity among radiologists,
  - in-depth analysis of every high dose examination compared to local DRLs and sometimes national DRLs, enabling appropriate investigations and, if needed, corrective action when local or national DRL values are consistently exceeded. Further optimisation should include a review of equipment performance and examination protocols, including settings used, procedure protocol, operator skill, and, for interventional techniques, procedure complexity.
- Periodically, ensure clinical-indication protocol harmonisation among modalities, re-estimate local DRL and optimise/improve practice where needed. If a new technology is implemented, re-evaluate local DRLs in collaboration with your medical physics expert.

## Summary and future perspective

DRLs should be viewed as a dynamic tool that is evolving to keep up with technology and clinical developments. DMS can play an important role in organising data needed for the implementation of this tool, allowing a frequent update of local DRLs. For the same anatomical region, the image quality needed depends on the clinical indication. For this reason, transition from anatomical-based DRLs to clinical indication-based DRLs is needed for national as well as local DRLs. Using local DRLs as a continuous quality improvement tool enables implementing a dose reduction culture by guiding radiologists, medical physicists and radiographers, towards a change of practice, to deliver the right dose for the right indication and achieve excellence in terms of quality and safety in medical imaging.

## Data Availability

Not applicable.

## Abbreviations

ALARA: As low as reasonably achievable
CTDI: CT dose index
DMS: Dose management systems
DRLs: Diagnostic reference levels
EC: European Commission
EFOMP: European Federation of Organizations for Medical Physics
EFRS: European Federation of Radiographer Societies
ESPR: European Society of Paediatric Radiology
ESR: European Society of Radiology
EUCLID: European Study on Clinical Diagnostic Reference Levels for X-ray Medical Imaging
FGIP: Fluoroscopically-guided interventional procedures
ICRP: International Commission of Radiological Protection
PiDRL: European Guidelines on Diagnostic Reference Levels for Paediatric Imaging
